# Cryptic SARS-CoV-2 lineage identified on two mink farms as a possible result of long-term undetected circulation in an unknown animal reservoir, Poland, November 2022 to January 2023

**DOI:** 10.2807/1560-7917.ES.2023.28.16.2300188

**Published:** 2023-04-20

**Authors:** Katarzyna Domańska-Blicharz, Bas B Oude Munnink, Anna Orłowska, Marcin Smreczak, Justyna Opolska, Anna Lisowska, Paweł Trębas, Wojciech Socha, Aleksandra Giza, Arkadiusz Bomba, Ewelina Iwan, Jerzy Rola, Marion Koopmans

**Affiliations:** 1 Department of Poultry Diseases, National Veterinary Research Institute, Puławy, Poland; 2 Department of Viroscience, Erasmus University Medical Centre, Rotterdam, the Netherlands; 3 Department of Virology, National Veterinary Research Institute, Puławy, Poland; 4 Department of Omics Analyses, National Veterinary Research Institute, Puławy, Poland

**Keywords:** SARS-CoV-2, mink, Poland

## Abstract

In late 2022 and early 2023, SARS-CoV-2 infections were detected on three mink farms in Poland situated within a few km from each other. Whole-genome sequencing of the viruses on two of the farms showed that they were related to a virus identified in humans in the same region 2 years before (B.1.1.307 lineage). Many mutations were found, including in the S protein typical of adaptations to the mink host. The origin of the virus remains to be determined.

The widespread circulation of severe acute respiratory syndrome coronavirus 2 (SARS-CoV-2) in humans on the one hand and the considerable number of susceptible wildlife hosts at the human–animal interface on the other hand poses a danger of reverse zoonotic spillover into animal populations. This includes wild animals, which could lead to the establishment of novel wildlife reservoirs [1,2]. Mink are especially susceptible and infections have been reported in North America and in several countries in Europe. 

Here, we report the detection of a cryptic SARS-CoV-2 lineage on two mink farms in late 2022 and early 2023 in Poland. The closest match was with lineage B.1.1.307 (GR/20B) viruses last detected in humans in late 2020 and early 2021, but the virus detected in mink had at least 40 nt changes, suggesting that it may originate from an unknown or undetected animal reservoir.

## Infection detection and information of the mink farms 

The monitoring system on Polish mink farms was launched in May 2020 and has been subject to various changes since then. Initially, it was carried out according to the relevant regulations of the Polish Ministry of Agriculture and Rural Development, taking into account scientific reports on SARS-CoV-2 infections in mink, later on also European Union (EU) Commission Decision 2021/788 [3,4]. From December 2021, all mink farms in Poland were tested for the presence of SARS-CoV-2 according to the scheme which stipulates sampling when disease symptoms or increased mortality were detected in the animals or when workers on the farm were SARS-CoV-2-positive (sample size designed to detect a 50% or 5% prevalence, respectively, with 95% confidence) [5]. The implemented system resulted in the identification of the first positive farm in January 2021, another 13 farms in the following months up to July 2022 [6,7]. These 14 positive farms were infected with four different SARS-CoV-2 variants belonging to eight different Phylogenetic Assignment of Named Global Outbreak (Pango) Lineages: 20B (two farms, B.1.1), Delta (21J, eight farms, AY.4, AY.43, AY.122, AY.126, B.1.617.2), Alpha (20I, one farm, B.1.1.7) and Omicron (21L, one farm, BA.2). For two farms, genome sequences were not obtained. The vast majority of mink SARS-CoV-2 infections were traced back to variants dominant in human samples during the COVID-19 epidemic in Poland, although one of the Alpha variants was detected many months after the epidemic’s peak [7]. The next three positive mink farms (Farms 14, 16 and 17) were identified between September 2022 and January 2023 in the same municipality and district (Table). We append details about the farms’ location in Supplementary Figure S1. 

**Table:** SARS-CoV-2 identification on three mink farms, Poland, September 2022–January 2023 (n = 23 isolates)

Farm	Date of sampling	Number of mink on the farm	Real-time RT-PCR		Isolate number	Cq range	Clade	Pango lineage
			Positive/tested	Cq range				
14	19 Sep 2022	8,650	2/15	33.4–33.9	NA			
16	16 Nov 2022	4,000	6/15	20.7–33.0	EPI_ISL_16811138	24.2	GR/20B	B.1.1.307 (probably new?)
					EPI_ISL_16811146	26.1		
					EPI_ISL_16811151	23,4		
					EPI_ISL_16811154	20.7		
17	18 Jan 2023	1,100	15/15	18.3–29.1	EPI_ISL_16994016	18.3		
					EPI_ISL_16994017	19.1		
					EPI_ISL_16994018	21.0		
					EPI_ISL_16994087	23.9		

Cq: quantification cycle; NA: not available; SARS-CoV-2: severe acute respiratory syndrome coronavirus 2.

Of 15 oropharyngeal swabs sampled on Farm 14 on 19 September, two tested positive by real-time RT-PCR with low virus concentrations (quantification cycle (Cq) values 33 and 34). On Farm 16, samples were taken on 16 November from 15 mink and six of them were SARS-CoV-2-positive with Cq values ranging from 20 to 26. The third farm (Farm 17) was identified on 18 January, and all 15 swabs tested positive with Cq values ranging from 18 to 29 (Table). 

The farms were located in a typical lowland agricultural area, with farmland interspersed with mixed and deciduous forests. The populations sizes were ca 8,650 mink (Farm 14), ca 4,000 mink (Farm 16) and ca 1,100 mink (Farm 17), and the farms were within an 8 km range of each other (see Supplementary Figure S1 for an aerial view of the region). A fourth farm in the same region has so far been negative. The three positive farms had tested negative in previous monthly screenings since 2021.

When positive, farms are per decision by the Ministry of Agriculture subjected to rigorous isolation and the animals are tested repeatedly (first sampling not earlier than after 30 days, and subsequently every 20 days) for the presence of SARS-CoV-2 until two consecutive rounds of negative testing [3,4]. Polish legislation mandates the culling of all animals on a farm if the mink mortality rate exceeds 10% or in case of mink-to-human transmission. This was not the case on these three positive farms. The mink on Farm 14 tested negative in the samplings following the positive test were pelted in the planned prescribed timeframe. Farm 16 turned out to test positive just before the pelting process, and animal pelting was done with modifications laid down in the Polish regulations [3]. The farm decided not to continue mink breeding in the following season. Samples (60 swabs) from mink on Farm 17 were tested twice, 30 and 50 days after the detection of SARS-CoV-2, and were negative.

## Molecular investigation

Whole genome sequencing was performed on all samples from Farm 14 and the four samples with the highest viral load from farms 16 and 17 as described [7]. Eight complete genomes were obtained (Table) and have been deposited in GISAID. The samples from Farm 14 had insufficient viral load for sequencing. Pango lineage classification showed that these viruses were part of the B.1.1.307 lineage [8]. Phylogeny based on all complete genome sequences of this lineage revealed that viruses from both farms formed a cluster and were most closely related to sequences collected from patients in the same region about 2 years earlier (Figure), but with more than 40 single nucleotide polymorphisms (SNPs). In Supplementary Table S4 we provide detailed information on changes in the mink’s SARS-CoV-2 genomes. This included amino acid substitutions W64L, F486L, N501T, T572I and S929I and a deletion of four amino acids at positions 140–143. The substitutions in positions 486 and 501 have previously been associated with circulation in mink [9].

**Figure:**
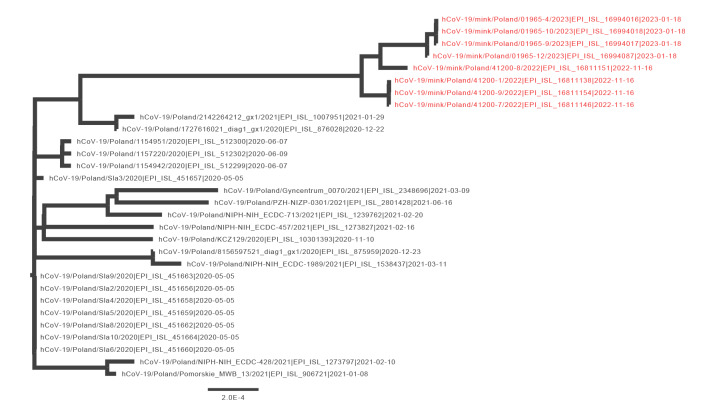
Phylogenetic tree of all SARS-CoV-2 whole genome sequences of lineage B.1.1.307 compared with isolates from mink, Poland, September 2022–January 2023 (n = 8)

SARS-CoV-2: severe acute respiratory syndrome coronavirus 2.

Sequences derived from the mink farms are depicted in red.

## Inspection carried out on infected farms

In order to find potential sources of introduction, a detailed investigation was done through interviews and site visits following the procedure described in Sikkema et al, which also defines the different types of barriers in place at mink farms [10]. The farm workers as well as the owners' families tested negative in real-time RT-PCR, making it unlikely that a chronic shedder introduced the virus to mink. Unfortunately, no serology was performed so we do not know if they had been infected in the past. All three farms have a (concrete) fence as a first barrier ca 1.8 m high and dug ca 30–40 cm deep into the ground around the farm site (Barrier 1). A visual inspection of this fence did not reveal any holes or potholes through which wild animals could enter. However, tall trees on both sides of the fence had branches which reached across, potentially providing a route for animals to enter the farm area [10]. Mink are kept in wire mesh cages (Barrier 3) which are sheltered from the top by a long roof set on poles without walls (no Barrier 2). At the entrance to the farms are corrugated metal gates fitted with a rubber seal at the very bottom (Supplementary Figure S2 contains photographs with examples of these structural barriers). 

Interviews conducted with staff and owners indicated the occasional presence of wild carnivores (martens) on the farms. In addition, visits of wild bird species were reported (see Supplementary Inventory Form S3 for a list of responses from the interviews). Feral cats were also observed on all farms, as evidenced by numerous fresh droppings. Three droppings were tested in February 2023 and were negative for SARS-CoV-2. Although the farm's employees did not report escaped mink, and the traps deployed for such animals were empty, it cannot be ruled out that animals may have escaped (see Supplementary Figure S3 for an example of a trap). There were no signs of bats on the farms.

## Discussion

Human-to-animal transmission of SARS-CoV-2 and reverse spillover is well documented worldwide [11-13]. After the culling of mink in Denmark and the Netherlands, Poland became the leading producer of mink in Europe and the second in the world after China, despite the major impact of the COVID-19 pandemic on mink production in Poland (a reduction from 350 farms in 2019 to 166 farms in March 2023). Such a decline in the farmed mink population was mainly caused by problems with the export of pelts and, most importantly, a decline in demand for fur around the world.

We describe the detection of a new, cryptic lineage of SARS-CoV-2 on two mink farms. These infections were detected 3 months apart, and the infected farms were in close proximity. The identified viruses were nearly identical and contained a number of mutations versus the Wuhan strain and human B.1.1.307 SARS-CoV-2, including F486L and N501T in the spike protein, which hint towards mink adaptation. Furthermore, the identified mink SARS-CoV-2 variant was most closely related to B.1.1.307 viruses detected in humans in different parts of Europe more than 2 years earlier. Since all mink farms in the region and also the farm workers and the owners' families have repeatedly tested negative, it is possible that the virus was introduced from some other, undetermined animal host where it may have been circulating undetected. Some of the mutations (486 and 501 in the spike) in the presented isolates have previously been found during prolonged circulation in mink, but involvement of other intermediate hosts like cats or other wild carnivores cannot be excluded and there was a clear opportunity for contact between wild animals and three inspected farm animals. One possibility is that the virus was introduced from free-living mink which have been found around many farms in past studies [14,15], but this hypothesis needs to be tested. It cannot be ruled out that infection of mink with such a virus occurred quite recently. The identified 40 nt differences from the nearest human virus is not a large and unexpected number, and it is likely that it was acquired during rapid evolution in mink over a shorter period of time.

The animals on the SARS-CoV-2-positive mink farms did not show signs of disease, which creates a possibility of independent viral evolution and may establish a source for future outbreaks with novel strains. Until now, spillback of this cryptic SARS-CoV-2 lineage has not been detected in the human population. The surveillance system for SARS-CoV-2 infections in the region should be strengthened by testing mink and humans on these farms more frequently, but also wild animals such as feral mink and cats and other carnivores such as martens, polecats or foxes should be tested molecularly and serologically.

## Conclusion

Despite a notable reduction in the number of mink farms, this type of production still exists in Poland. The monitoring of SARS-CoV-2 infections, which has been introduced for several years, has made it possible to detect 14 positive farms, and the identified viruses were similar to those circulating in humans at the time. However, on the two most recently positive farms, the detected virus was altered to form a new cryptic Pango lineage. Conducting routine surveillance for SARS-CoV-2 on mink farms seems necessary, since the animals described here were asymptomatic, and the viruses would have gone undetected without mandatory viral surveillance. It seems that relying only on passive surveillance in response to symptomatic outbreaks could result in many cases being overlooked.

## Supplementary Data

643000 Supplement
