# Supplementary material for: Cryptic SARS-CoV-2 lineage identified on two mink farms as a possible result of long-term undetected circulation in an unknown animal reservoir, Poland, November 2022 to January 2023
Source: Euro Surveill. 2023 Apr 20;28(16):2300188. doi: 10.2807/1560-7917.ES.2023.28.16.2300188 (PMC10283451; doi:10.2807/1560-7917.ES.2023.28.16.2300188)
Supplement: Supplement [file 23-00188_DOMANSKA-BLICHARZ_Supplement.pdf]

Supplementary materials

S1. Aerial image of an area with infected mink farms (yellow circles – infected farm, red circle – the fourth negative farm, yellow line – scale 1 km)

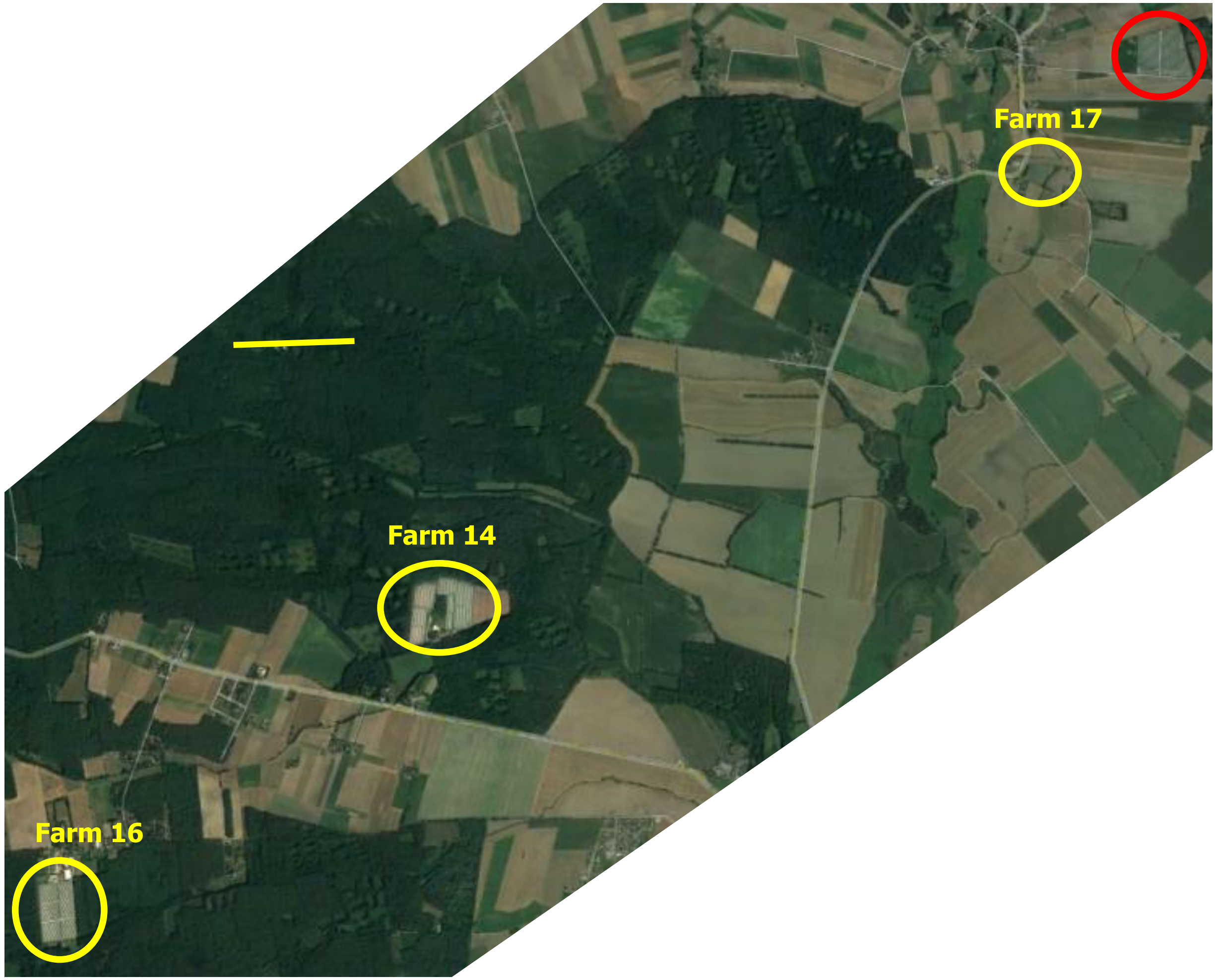

S2. Photo’s of mink farm in Farm 17

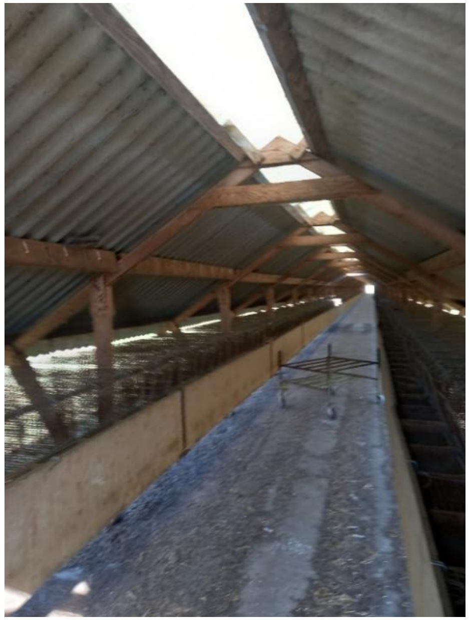

Mink hall (with Barrier 3 seen)

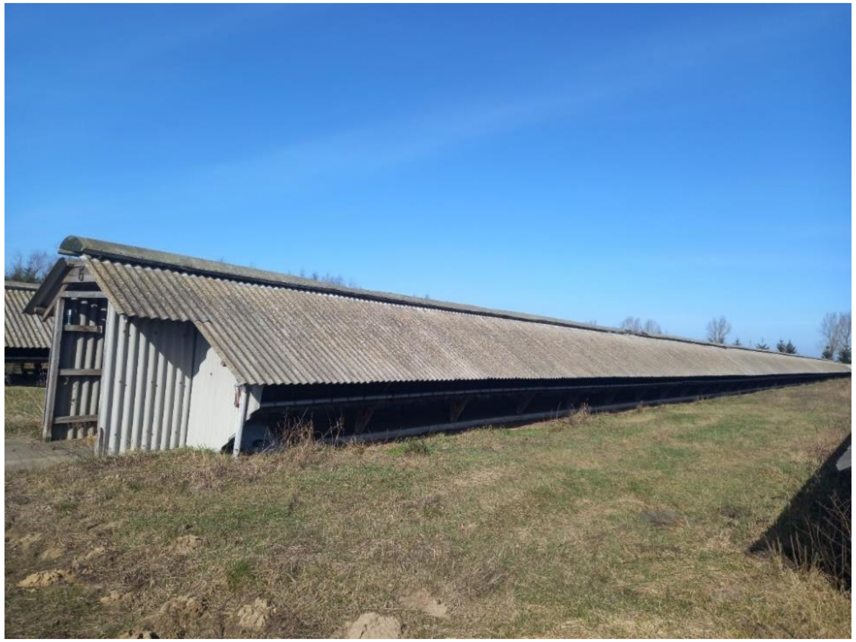

Mink sheds (no Barrier 2 – no walls of sheds)

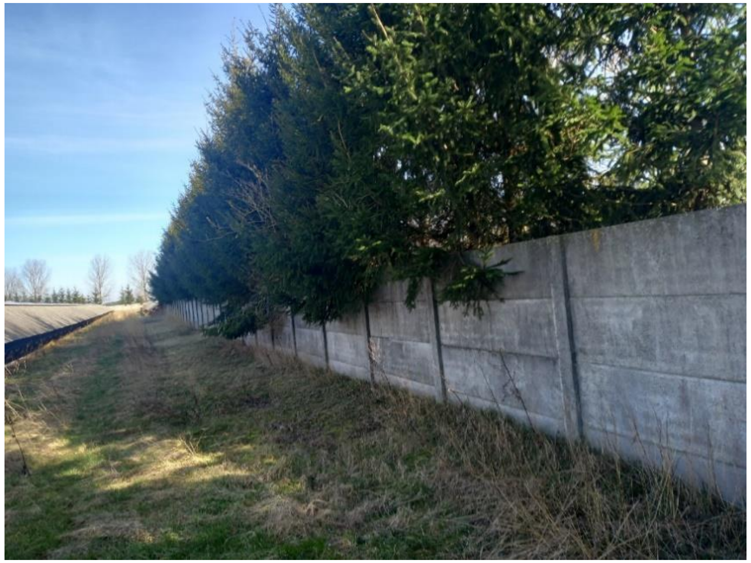
Concrete fence with visible trees (Barrier 1)

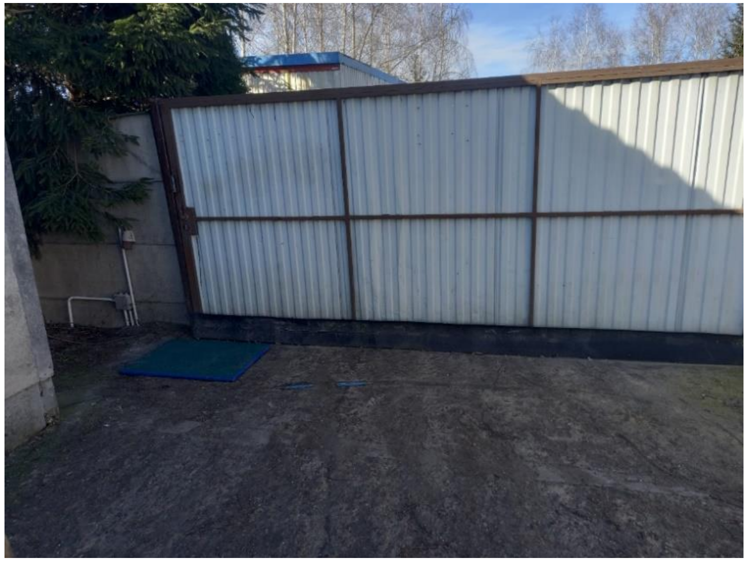
Corrugated sheet metal gate secured from below with rubber seal, adjacent trees visible (Barrier 1)

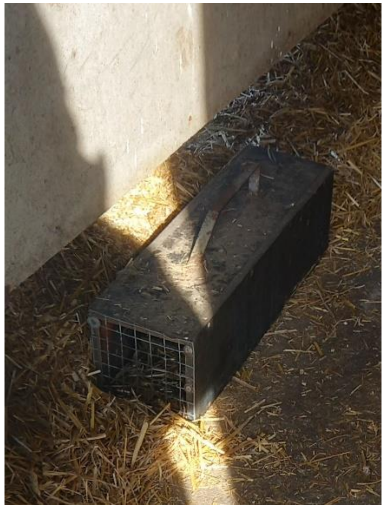
Traps for escaped mink

S3. Infected mink farms inventory form

Birds present on farms:
Species observed: house sparrow, pigeon
Number of observed nests of those species: sparse

Opportunities for foxes & mustelids to access farms:
Fence:
Fencing type: concrete
Height: 1,8 m
Electric fencing/wiring? No
Digging prevention type: fencing about 30-40 cm under ground
Gates:
Port type: corrugated metal gates fitted with a rubber seal at the very bottom
Height: 1,8 m
Other:
Wildlife trails around terrain: Probably yes
Impression where animals could enter (photos above): the presence of numerous, tall and spreading spruce trees just behind/inside the fence, whose branches reached into the farm/outside area (bridging the farm area and the outside environment)
Weakest link(s):
Are traps available for escaped minks: yes
Stray/house cats present: yes
Surroundings of the farm/Small landscape elements present: the presence of numerous, tall and spreading spruce trees

Foraging options for bats:
Type: Old sheds and additionally two sealed cold storage halls, the only open building is a washer but with no signs of bats, most of the farm currently unused - no signs of bats or other animal
How many M^2 of this type: 10 450
Type of roof: sheet pile profile
Color roof: grey
Accessibility of bats: ends open
Height of ridge to cages (flight space): 1,5 m
Height of ground to under cages (flight space): 2,5 m
Ventilation: air circulation
Light present: Yes
Can you find traces of bats near houses: No

Impression: can there be a maternity roost: Rather not
Connectivity in the landscape: Moderate
Manure storage solid manure: Open
General impression w.r.t. bats: May be sitting in trees but it's hard to judge

S4. Sequence changes within SARS-CoV-2 in mink on Polish farms

**Structure**		**ORF1a**															**ORF1b**						
**Aa**		**90**	**315**	**988**	**1188**	**1920**	**2210**	**2495**	**2685**	**3338**	**3371**	**3579**	**3606**	**3753**	**3934**	**4177**	**314**	**1643**	**1808**	**2162**	**2294**	**2530**	**2557**
**Virus**																							
**Wuhan**		A	M	V	S	Y	C	T	P	L	P	T	L	F	M	G	P	A	E	P	S	D	K
**EPI_ISL_876028**		A	I	G	S	Y	C	T	P	L	P	T	L	F	I	G	L	V	E	P	N	D	K
**EPI_ISL_1007951**		A	I	G	S	Y	C	T	P	L	P	T	L	F	I	G	L	A	E	P	S	D	K
**Farm 16**	**EPI_ISL_16811138**	V	I	G	L	Y	C	I	T	F	S	I	F	V	I	E	L	A	E	S	S	D	K
	**EPI_ISL_16811146**	V	I	G	L	Y	C	I	T	F	S	I	F	V	I	E	L	A	E	S	S	D	K
	**EPI_ISL_16811151**	V	I	G	L	Y	C	I	T	L	S	I	F	F	I	E	L	A	D	S	S	E	K
	**EPI_ISL_16811154**	V	I	G	L	Y	C	I	T	F	S	I	F	V	I	E	L	A	E	S	S	D	K
**Farm 17**	**EPI_ISL_16994016**	V	I	G	L	C	F	I	T	L	S	I	F	F	I	E	L	A	D	S	S	D	R
	**EPI_ISL_16994017**	V	I	G	L	C	F	I	T	L	S	I	F	F	I	E	L	A	D	S	S	D	R
	**EPI_ISL_16994018**	V	I	G	L	C	F	I	T	L	S	I	F	F	I	E	L	A	D	S	S	D	R
	**EPI_ISL_16994087**	V	I	G	L	Y	F	I	T	L	S	I	F	F	I	E	L	A	D	S	S	D	R

**Structure**		**S**											**M**	**N**			**ORF3a**	**ORF7b***	**ORF8****
**Aa**		**64**	**95**	**140**	**141**	**142**	**143**	**486**	**501**	**572**	**614**	**929**	**190**	**119**	**203**	**204**	**93**	**13**	**42**
**Virus**																			
**Wuhan**		W	T	F	L	G	V	F	N	T	D	S	D	A	R	G	H	F	Y
**EPI_ISL_876028**		W	I	F	L	G	V	F	N	T	G	S	D	V	K	R	H	F	Y
**EPI_ISL_1007951**		W	I	F	L	G	V	F	N	T	G	S	D	V	K	R	H	F	Y
**Farm 16**	**EPI_ISL_16811138**	L	T	-	-	-	-	**L**	**T**	I	G	I	D	A	K	R	Y	-	.
	**EPI_ISL_16811146**	L	T	-	-	-	-	**L**	**T**	I	G	I	D	A	K	R	Y	-	.
	**EPI_ISL_16811151**	L	T	-	-	-	-	**L**	**T**	I	G	I	D	A	K	R	Y	-	.
	**EPI_ISL_16811154**	L	T	-	-	-	-	**L**	**T**	I	G	I	D	A	K	R	Y	-	.
**Farm 17**	**EPI_ISL_16994016**	L	T	-	-	-	-	**L**	**T**	I	G	I	E	A	K	R	Y	-	.
	**EPI_ISL_16994017**	L	T	-	-	-	-	**L**	**T**	I	G	I	E	A	K	R	Y	-	.
	**EPI_ISL_16994018**	L	T	-	-	-	-	**L**	**T**	I	G	I	E	A	K	R	Y	-	.
	**EPI_ISL_16994087**	L	T	-	-	-	-	**L**	**T**	I	G	I	E	A	K	R	Y	-	.

*frame shift from codon 13, **stop codon
Amino acids changes found only in mink viruses gray-shaded, bold – mink-specific substitution
